# The FIFA Women’s World Cup in Germany 2011 – A practical example for tailoring an event-specific enhanced infectious disease surveillance system

**DOI:** 10.1186/1471-2458-12-576

**Published:** 2012-07-31

**Authors:** Anja Takla, Edward Velasco, Justus Benzler

**Affiliations:** 1 Department of Infectious Disease Epidemiology, Robert Koch Institute, Berlin, Germany; 2 Postgraduate Training for Applied Epidemiology (PAE), Berlin, Germany; 3 European Programme for Intervention Epidemiology Training (EPIET), European Centre for Disease Prevention and Control, Stockholm, Sweden

## Abstract

**Background:**

Mass gatherings require a decision from public health authorities on how to monitor infectious diseases during the event. The appropriate level of enhanced surveillance depends on parameters like the scale of the event (duration, spatial distribution, season), participants’ origin, amount of public attention, and baseline disease activity in the host country. For the FIFA Men’s World Cup 2006, Germany implemented enhanced surveillance. As the scale of the FIFA Women’s World Cup (June 26 – July 17, 2011) was estimated to be substantially smaller in size, visitors and duration, it was not feasible to simply adopt the previously implemented measures. Our aim was therefore to develop a strategy to tailor an event-specific enhanced surveillance for this smaller-scale mass gathering.

**Methods:**

Based on the enhanced surveillance measures during the Men’s Cup, we conducted a needs assessment with the district health authorities in the 9 host cities in March 2011. Specific measures with a majority consent were implemented. After the event, we surveyed the 9 district and their corresponding 7 state health authorities to evaluate the implemented measures.

**Results:**

All 9 district health authorities participated in the pre-event needs assessment. The majority of sites consented to moving from weekly to daily (Monday-Friday) notification reporting of routine infectious diseases, receiving regular feedback on those notification reports and summaries of national/international World Cup-relevant epidemiological incidents, e.g. outbreaks in countries of participating teams. In addition, we decided to implement twice-weekly reports of “unusual events” at district and state level. This enhanced system would commence on the first day and continue to one day following the tournament. No World Cup-related infectious disease outbreaks were reported during this time period. Eight of 9 district and 6 of 8 state health authorities participated in the final evaluation. The majority perceived the implemented measures as adequate.

**Conclusions:**

Our approach to tailor an event-specific enhanced surveillance concept worked well. Involvement of the participating stakeholders early-on in the planning phase secured ownership of and guaranteed support for the chosen strategy. The enhanced surveillance for this event resulted as a low-level surveillance. However, we included mechanisms for rapid upscaling if the situation would require adaptations.

## Background

Mass gatherings can be accompanied by a number of health risks – especially the increase in population density, the import and export of unusual pathogens, and temporary changes in services like provisional food stalls, all of which can increase the possibility for infectious disease spread [1]. As a result, public health authorities have begun to develop strategies for prevention and response. For example, they enhance routine surveillance and/or introduce additional syndromic surveillance during mass gatherings to timely detect and react on adverse health events [2-6]. The following parameters may influence the appropriate level of enhanced surveillance for mass gatherings: (a) number of participants, (b) duration of the event, (c) its spatial distribution, (d) origin of participants, (e) level of infectious disease activity in the host country, and (f) amount of public attention.

Recent consensus prescribes that enhanced surveillance systems should be in place for big-scale international mass gatherings, and they have been routinely implemented during the past years. Most previously published descriptions of enhanced surveillance concern recurring large-scale mass gatherings like the annual pilgrimage to Mecca [6], or sport events like the Olympics or FIFA Men’s World Cups [1,3-5,7-13]. As these mass events are usually predictable in dimension, structure, and amount of public attention, publications on experiences with the implemented surveillance measures can be of great assistance for new host countries to set up surveillance for their event. However, deciding on an adequate surveillance strategy for a medium-scale mass event can be difficult if (a) an event is new, (b) one or more of the influencing parameters are unclear or differ greatly to previous occasions, and (c) no prior experience with similar events is available.

For 2011, Germany was chosen to host the 6^th^ FIFA Women’s World Cup. Even though men’s football is the most popular sport in Germany, it was unclear to what extend the Women’s World Cup would benefit from the men’s sports popularity in terms of number of tickets sold and visitors attending public fan festivals. Publications on surveillance concepts during previous FIFA Women’s World Cups were not available, but it was documented that the tournaments had fluctuated considerably in size (115.000 - 1.2 million tickets sold) over the past years [14].

Since 2001, Germany has a well functioning routine electronic reporting system for notifiable infectious diseases (called “SurvNet” - developed by the Robert Koch Institute (RKI) - and a number of comparable commercial software products) [15,16] that transmits surveillance data from the >350 district health authorities via the corresponding 16 federal state authorities to the RKI, the National Public Health Institute. The German Infection Protection Act from 2001 determines which infectious diseases are notifiable by physicians and laboratories to the district health authorities [17]. Notifications of cases and outbreaks are weekly transmitted from the district health authorities to the state health authorities and on to the RKI. For a small number of pathogens, e.g. imported diseases like malaria with no potential for further local transmission, laboratories and/or physicians transmit notifications directly to the RKI. In general, responsibility for surveillance and containment actions solely lies with the district health authorities.

Six months prior to the Women’s World Cup in Germany, only limited specific information on the tournament was available: the games were to take place over 3 weeks from June 26 to July 17; a total of 16 teams had qualified, 10 coming from developed countries (Germany, England, France, Norway, Sweden, Australia, New Zealand, Canada, United States, and Japan) and 6 from developing countries (Equatorial Guinea, Nigeria, North Korea, Brazil, Columbia, and Mexico); games were to be carried out in 9 cities (located in 7 of the 16 federal states) all over Germany [18]; and approximately 900,000 tickets were going into public sale [19]. However, ticket sales remained slow in the pre-phase of the event and public attention was low to moderate.

Five years prior to the FIFA Women’s World Cup, Germany had hosted the men’s competition, which had taken place in 12 cities over a time period of 4 weeks with a total of 32 teams from 6 continents. Around 3 million stadium tickets had been sold, and another estimated 21 million people attended public viewing sites and fan festivals [1]. For the event, health authorities enhanced the pre-existing surveillance system by e.g. acceleration of data transmission and introduction of an additional free-text reporting system for relevant public health events; additional syndromic surveillance was not introduced. The chosen strategy hereby proved to be an effective approach [1,5,20].

Positive experiences with enhanced surveillance during the Men’s Cup led to the decision to likewise enhance surveillance without a syndromic component for the Women’s Cup. As the women’s event was—in comparison to many aspects—anticipated to be considerably smaller, we deemed re-implementation of the enhanced surveillance measures from the Men’s Cup as inappropriate and thus needed to newly determine an adequate event-specific surveillance level. We therefore aimed to introduce an approach for tailoring an adequate enhanced surveillance for smaller-scale mass gatherings, using the example of the FIFA Women’s World Cup 2011 in Germany.

## Methods

Our tailoring of an event-specific enhanced surveillance system consisted of 3 steps: first, we conducted a needs assessment with the affected district health authorities and assessed the scale of the event. The second step consisted of the actual implementation of the surveillance measures. In the third step, we carried out an evaluation of the implemented measures with the involved stakeholders.

### Pre-event measures

#### Needs assessment of the district health authorities

In March 2011, we performed the needs assessment with the 9 district health authorities in the 9 host cities. We conducted phone-based interviews with the person responsible for the infectious disease surveillance during the World Cup, using a semi-structured questionnaire. Questions were asked on the district health authority’s state of preparation, existing resources in terms of infrastructure and emergency preparedness, and perceived need for specific surveillance measures. In addition, we asked the district health authorities if they were interested in participating in a course on “Advanced Management of Biological Threats (AMBIT),” offered by the German Federal Institute for Biological Security as part of the World Cup preparation.

The list of suggested specific surveillance measures was based on measures implemented during the FIFA Men’s World Cup 2006 in Germany [1,5]. According to expert opinion and resource constraints certain measures that seemed appropriate only for large-scale mass gatherings (e.g. establishment of a permanent crisis room) were dropped. District health authorities could select from that list, but also opt for measures that were not stated. The consented upon measures could be further supplemented by measures that were deemed important by RKI experts experienced with mass gathering events.

#### Assessment of the event scale

To assess the scale of the event, we contacted the German Football Association (DFB) to obtain numbers on domestic and foreign ticket sales. Furthermore, we spoke to the tourist offices in the host cities to learn about hotel pre-bookings associated with the event.

### Implementation and event surveillance

Implementation of enhanced surveillance measures was based on a majority consensus of the 9 district health authorities. Enhanced surveillance was to be implemented from the first day of the event to 1 day after its end. Besides the district health authorities, their corresponding state health authorities were also involved in surveillance and communication measures during the event. Monitoring of transmitted notifications from the health authorities in each host city was performed at the RKI by daily comparison to the corresponding figures of the previous week and running algorithms to filter for events tagged as World Cup-associated.

### Post-event evaluation of the implemented system

On July 25, 2011, we emailed a structured questionnaire to the 9 district and 7 state health authorities to evaluate the implemented surveillance activities in terms of feasibility and appropriateness. Moreover, participants were asked about the value of the pre-event needs assessment and to comment on the specific measures and overall appropriateness of the implemented system.

### Ethical approval

Our study did not involve any experimental research on humans. The RKI has the legal authority to collect and analyze the national statutory infectious disease surveillance data (based on the German Infection Protection Act 2001) reported by district and state health authorities. Informed consent or approval of an ethics committee was not required, as the individual surveillance data used was anonymous and retrieved from this statutory surveillance system. Participation of the district and state health authorities, respectively, in the pre-event needs assessment and final evaluation of the surveillance concept was voluntary.

## Results

### Pre-event measures

#### Needs assessment of the local health authorities

All 9 district health authorities took part in the needs assessment. Four reported to have an on-call duty for weekends and nights on a routine basis, an additional 3 informed us that they were planning to install an on-call duty for the duration of the World Cup. All 9 stated to have operation schedules for infectious disease emergencies, updated phone-lists, and rapid access to personal protective equipment. Six were interested in participating in the AMBIT course. A majority of district health authorities agreed with 5 of the 8 proposed surveillance measures (Table 1). Due to financial constraints and existing or to be implemented on-call duty for weekends the majority of district health authorities requested transmission of infectious disease notifications only Monday-Friday.

**Table 1:** Proposed enhanced surveillance measures for the FIFA Women’s World Cup 2011 in Germany and number of district health authorities (n = 9) in agreement with the measure, pre-event needs assessment, March 2011

**Surveillance measure**	**No. of district health authorities in agreement (n = 9)**
WITH majority agreement	
Pre-event creation of network among public health stakeholders	5
Daily (Monday to Friday) transmission of infectious disease notifications	5
Regular feedback report of districts’ infectious disease notifications	6
Regular summary report of World Cup-relevant national/international epidemiological events*	8
Phone conferences among stakeholders (on demand)	6
WITHOUT majority agreement	
Pre-event meeting of public health stakeholders	4
Pre-event reminder of laboratories/physicians on infectious disease reporting duty	2
Daily (Monday to Friday) reports from district health authorities of unusual high numbers of infectious diseases/syndromes^#^	3

* defined as epidemiological events (e.g. outbreaks) in Germany or countries with a participating team or countries with >100 visitors.

^#^ defined as e.g. unusual high number of pneumonia cases in the district’s hospital(s).

To supplement the consented upon measures, the RKI decided to additionally implement a “report of unusual events” from district and state health authorities. There were no pre-specified criteria to define such an event. The report was to be used for any incident that the health authorities thought relevant for the other World Cup stakeholders. This could include e.g. suspected but not yet confirmed infectious disease cases in the district or outbreaks located in other districts within the state. Furthermore, the RKI decided on a twice-per-week (Monday and Thursday) reporting schedule for the regular feedback report of the districts’ notifications, the “report of unusual events” from district and state health authorities, and the “regular summary of World Cup relevant events”. Cases linked to the World Cup (defined as team members, support staff or spectators including travels to or from the stadium and having visited any World Cup-associated event like a game or fan zone) were to be tagged by the district health authorities in the SurvNet system with a specific “World Cup 2011” flag. In addition to the daily infectious disease notifications and the “reports of unusual events”, the RKI would use the “World Health Organization Outbreak Verification List” (GOARN WHO), the “Rapid Alert System for Food and Feed”, the “European Early Warning and Surveillance System”, the Center for Travel Medicine (“CRM Centrum für Reisemedizin”), “Promed”, “EuroSurveillance”, the “CDC Morbidity and Mortality Weekly Report”, “Google Health Map”, and press releases as sources for the regular summary of national and international World Cup relevant events.

#### Assessment of the event scale

By the beginning of June, we were informed by the DFB that approximately 520,000 tickets had been sold, of those <5 % outside of Germany, with the vast majority going to the United States, France, United Kingdom, Sweden, Switzerland, Canada, and Austria. Tourist offices in the host cities reported no hotel pre-bookings for the event.

### Implementation and event surveillance

Prior to the event, the RKI organized a 2-day AMBIT course (June 14–15, 2011), that was directed at the district and state health authorities involved in the World Cup surveillance; 6 of the district and 3 of the state health authorities participated.

The following measures that a majority of district health authorities had consented upon and their RKI supplements were then implemented and put into practice from June 27 to July 18, 2011:

1. Pre-event collection and distribution of stakeholders’ contact details

2. Daily (Monday to Friday) transmission of infectious disease notifications from district via state level to the RKI

3. Using the tag "World Cup 2011" for World Cup-associated infectious disease cases in daily transmission

4. Twice-per-week reports of unusual events (including null reporting) from district health authorities to the RKI via the corresponding state health authority

5. Twice-per-week report (Monday and Thursday) of the RKI to district and state health authorities and ministry of health including

feedback report of daily district infectious disease reporting

feedback report of “reports of unusual events” from districts and state health authorities

summary report of World Cup relevant national/international epidemiological events

6. Phone conferences among stakeholders (on demand)

All district health authorities with one exception participated for the entire monitoring period in the daily infectious disease reporting: the district and state health authorities of one host city decided to stop all enhanced surveillance measures on July 7, as they were only hosting the opening game on June 26. Up to July 7, the 9 participating district health authorities transmitted a total of 35 of the expected 36 (97 %) reports on “unusual events” during that time period; for the 7 state health authorities, a total of 25 of the expected 28 (89 %) reports were sent in. After July 7 with 8 district and 6 state health authorities contributing, participation was 20/24 (83 %) and 16/18 (89 %) expected reports for the district and state health authorities, respectively.

We found no unusual increase of notifiable disease reporting in the weekday notification reports of the district health authorities compared to districts’ notification numbers from the previous week. Using the automated algorithm to extract the World Cup related notifications, none of the incoming reports via SurvNet was labelled with the “World Cup 2011” tag. Via the direct notification route from laboratories and/or physicians to the RKI, we received 1 report of a malaria case in a 14 year old girl from Kenya that came as a visitor for the World Cup event. In the “report of unusual events”, district and state health authorities reported 2 confirmed measles cases among participants of a football camp in Italy, with participants coming from another 7 German districts; and a *Salmonella* Bovismorbificans outbreak with 13 confirmed and 4 suspected cases in the state of Berlin. None of these outbreaks could be associated with the World Cup.

### Post-event evaluation of the implemented system

Eight of the 9 district health authorities and 5 of the 7 state health authorities took part in the post-event evaluation. Six district health authorities and 4 state health authorities regarded the pre-event needs assessment as valuable or very valuable, 1 district health authority as not valuable, and 1 each answered “don’t know”. A detailed evaluation of the implemented surveillance measures is listed in Table 2. One state health authority rated the surveillance concept as too extensive, since in their opinion the event does not require enhanced surveillance, and also as too limited, as all district health authorities (especially those bordering the host site district) should be integrated in the concept. Another state health authority also mentioned the lack of integration of bordering districts; in addition, 1 state health authority would have preferred to have weather forecast data provided in the summary report.

**Table 2:** Post-event evaluation of implemented enhanced surveillance measures during the FIFA Women’s World Cup in Germany, by involved district and state health authorities, July 2011

	**District health authorities (n = 8)**	**State health authorities (n = 6)**
Pre-event establishment of stakeholder network		
very important/important	6	4
less important/unimportant	2	2
Pre-event AMBIT course		
felt better prepared for potential threats	5	3 (*2)
not participated	2	1
Daily reporting of infectious diseases		
feasible	7	6
appropriate	5	5
Reporting of unusual events		
feasible	8	6
appropriate	6	3 (*2)
Feedback/summary report		
helpful	6	4 (*1)
appropriate regarding the structure	6 (*1)	5 (*1)
appropriate regarding the frequency	6	4
appropriate regarding the sources	7 (*1)	4 (*1)
Overall enhanced surveillance concept		
too limited	0	2†
adequate	5	3
too extensive	3	2†

* number of participants answered "don't know".

^†^ 1 state health authority answered too limited and too extensive.

## Discussion

We approached the task of tailoring an adequate enhanced surveillance for a smaller-scale mass gathering by first conducting a needs assessment with the involved stakeholders, followed by developing the enhanced surveillance components according to our findings. The strategy proved a successful method as shown by the stakeholders’ evaluation of the concept.

The needs assessment hereby served several purposes. First, it had the aim to encourage and support early preparations for the event by district health authorities. Second, it intended to actively involve the district health authorities in the planning process to create a system that was feasible and supported at the district health level. Third, it intended to secure district health authorities’ ownership and participation, and to guarantee their support of the chosen strategy. The post-event evaluations affirmed the acceptance of our approach; a majority among district health authorities rated the needs assessment as valuable and the overall surveillance strategy as adequate. Furthermore, participation rates of district health authorities in the surveillance measures were high throughout the tournament. The rates decreased a bit towards the second half of the competition, however this can be explained by the transition from group to play-off phase: with the start of the quarter-finals (July 9), 7 host cities were still involved; this number was then further reduced to 4 cities from the beginning of the semi-finals (July 13).

Creating a stakeholders network in the pre-event phase proved very helpful, as communication lines and familiarity with contact persons could be established. Consistent with our observations, this measure was positively evaluated across a majority of stakeholders. Even though a majority was not in favour of a pre-event meeting, many of the stakeholders met during the AMBIT course that took place two weeks before the beginning of the tournament. As levels of experience with mass gatherings were heterogeneous among the district health authorities, we believe that a pre-event meeting could have further facilitated knowledge transfer among the participating health authorities. However, most of the district health authorities stated time or financial constraints as reason for voting against such a meeting.

Though a majority was not in favour of reports on unusual events, we nevertheless decided to implement this measure. Based on experiences during the Men’s World Cup in Germany, Schenkel et al. had stated that “introducing an additional, sensitive, non-case definition-based written report system was overall beneficial” and recommended “additional reporting systems that are flexible and not bound to case-definitions, provided that at least one case-definition system or syndrome-based system is in place” [5]. The report on unusual events was intended as a very sensitive tool and a rapid, informal, low-threshold way of communicating noticeable incidents. As we did not include bordering districts in the enhanced surveillance activities, this was furthermore meant as an informal communication device for the state health authorities to inform about important events in the region that could have a potential impact on the World Cup. In the post-event evaluation, the vast majority of district and state health authorities regarded the measure as feasible and a majority as appropriate.

Based on our needs assessment and the general parameters that influence the intensity of enhanced surveillance, we decided for a low-level enhanced surveillance during the Women’s World Cup. However, we had mechanism in place for rapidly scaling up, if necessary, e.g. to increase the frequency of summary reports from twice-per-week to daily or to quickly establish a crisis room. A realistic example that would have required a rapid scaling up could have been the Shiga-toxin producing *Escherichia* coli O104:H4 outbreak in Germany [21], if first cases had occurred during the World Cup instead of May 2011.

Our approach had some limitations. The evaluation of the implemented measures could have been biased in two ways. First, answers in the evaluation by the district and state health authorities could have been influenced by trying to give socially desirable responses (social desirability bias). Second, with no severe events having occurred, measures could have been retrospectively evaluated as not necessary or inappropriate. We tried to address this bias by explicitly asking for an evaluation with the following prerequisite: “If a similar mass gathering event like the Women’s World Cup 2011 was to take place again and you as a state/district health authority were to participate in the same way, how would you rate, overall, the implemented measures of the enhanced surveillance strategy?”

Despite these potential biases, the foremost intention of our publication is to present a strategy on how to tailor an event-specific surveillance system – not to propose a comprehensive, ready-to-use enhanced surveillance system that should be translated directly to mass gathering events in other countries or surveillance systems. We believe that our approach holds valuable suggestions for health authorities in charge of enhanced surveillance in future smaller-scale mass gatherings, and that some of our specific implemented measures might be helpful templates for other prospective health authorities to consider.

## Conclusions

Involving the participating stakeholders early-on in the planning phase for tailoring an event-specific enhanced surveillance system secures ownership and later participation in the actual surveillance measures. Conducting a needs assessment is thereby a helpful tool to establish communication lines and to exchange ideas in the preparation phase. As smaller-scale mass gatherings often include a manageable number of stakeholders, a pre-event needs assessment involving all of them is usually feasible. Implemented measures should be based on a consensus of the participating stakeholders, however, if necessary, be supplemented by measures considered essential by mass gathering experts. Furthermore, enhanced surveillance systems for smaller-scale mass gatherings should allow for situation-dependent adaptations and include mechanisms for rapid up- or downscaling if required. A post-event evaluation of the implemented measures allows stakeholders to share their experiences and views, and helps to further improve the toolbox for authorities in charge of future enhanced surveillance systems.

## Competing interests

The authors declare that they have no competing interests.

## Authors’ contributions

AT participated in the design, coordination and analysis of the study and drafted the manuscript. EV participated in the design and coordination of the study and helped to draft the manuscript. JB conceived of the study, and participated in its design and coordination and helped to draft the manuscript. All authors read and approved the final manuscript.

## Pre-publication history

The pre-publication history for this paper can be accessed here:

http://www.biomedcentral.com/1471-2458/12/576/prepub
